# Quack Medicines

**Published:** 1869-07

**Authors:** 


### Quack Medicines.

Our Legislature has been considering the propriety of passing a bill, providing for the inspection of patent medicines, by a commission appointed for that purpose, whose duty shall be to report the ingredients of every patent medicine offered in the shops for sale.

If such a bill could be passed, it would doubtless have a wholesome effect upon the constitutions of our people, who swallow immense quantities of such miserable stuff every year.

